# ENflorA: a flexible tool for the bulk submission of next generation sequencing data to the European Nucleotide Archive

**DOI:** 10.1186/s12859-026-06714-7

**Published:** 2026-10-21

**Authors:** Antonio Alfaro de Prado Martín, Francisco Fajardo-Gutiérrez, Astrid de Mestier, Katja Reichel

**Affiliations:** 1 Institut für Biologie, Systematische Botanik und Pflanzengeographie, Freie Universität Berlin, Altensteinstraße 6, 14195 Berlin, Germany; 2 Botanischer Garten und Botanisches Museum Berlin, Freie Universität Berlin, Königin Luise-Straße 6-8, 14195 Berlin, Germany

**Keywords:** ENA, FAIR data, File transfer protocol, INSDC, High-throughput sequencing, Plastome, Submission

## Abstract

**Background:**

With the advancement of sequencing technologies and increasing quality of assembly and annotation tools, the amount of biological sequence data produced by research grows rapidly. To keep this data accessible for potential re-analysis, it is typically stored in public online databases, such as the European Nucleotide Archive (ENA). However, the increase in data entails an increase in time needed for metadata curation and upload. We propose a tool to simplify and automatize the upload of experimental and metadata to public repositories.

**Results:**

We developed ENflorA, a set of scripts by which sample, sequence read and (annotated) assembly data can be uploaded to ENA in a simple and standardized manner. Our software allows easy metadata entry via spreadsheets, is suitable for batch upload of data from different sources, and can be fully integrated in bioinformatic workflows. It is platform independent and can be run natively on High Performance Computers, integrates an lftp upload option for big files and a test upload option.

**Conclusions:**

By easing the ENA submission process, ENflorA contributes to making high-throughput sequencing data findable, accessible, interoperable and reusable.

**Supplementary Information:**

The online version contains supplementary material available at https://doi.org/10.1186/s12859-026-06714-7.

## Background

High-throughput sequencing technology has unlocked access to realms of new data from both human and non-human genomes, creating entirely new fields of research, particularly in biodiversity genomics [1, 2]. While per-base sequencing prices continue to fall [3], the overall cost and effort involved in producing this data, as well as the wealth of information contained in it, thoroughly justify its long-term storage for potential reuse. Beside the need for dedicated infrastructure to store petabyte-sized amounts of digital DNA sequences, data stewardship itself increasingly becomes a bottleneck: to make data findable, accessible, interoperable and reusable (FAIR principles [4]), it needs to be well documented with associated metadata, uploaded to a freely accessible, searchable online database, and formatted in accordance with existing data standards. As also the sequencing projects continue to grow [1, 2, 5], these time-consuming, intertwined tasks can no longer be managed “by hand”, but need automation, both to minimize errors and to be achievable within a set time frame [6, 7].

Public online databases, allowing researchers to upload/download (DNA) sequences and associated metadata, stored in standardized formats which facilitate searches and reuse, are a cornerstone of modern genetic research. In 1982, the International Nucleotide Sequence Database Collaboration (INSDC) was founded as a joint global effort to store this type of data and to make it available to researchers worldwide [8]. This major effort to coordinate the global exchange and long-term stewardship of nucleotide sequence data across partner archives, including the European Nucleotide Archive (ENA [9]), GenBank (NCBI [10]), and the DNA Data Bank of Japan (DDBJ [11]), has facilitated thousands of research projects and continues to ensure their repeatability, and thus scientific soundness. By regular mirroring of data between geographically independent infrastructure, the INSDC to date remains the safest standard for global research, though it is not the only DNA sequence “vault” (e.g. China National GeneBank CNGB [12]).

**Fig. 1:**
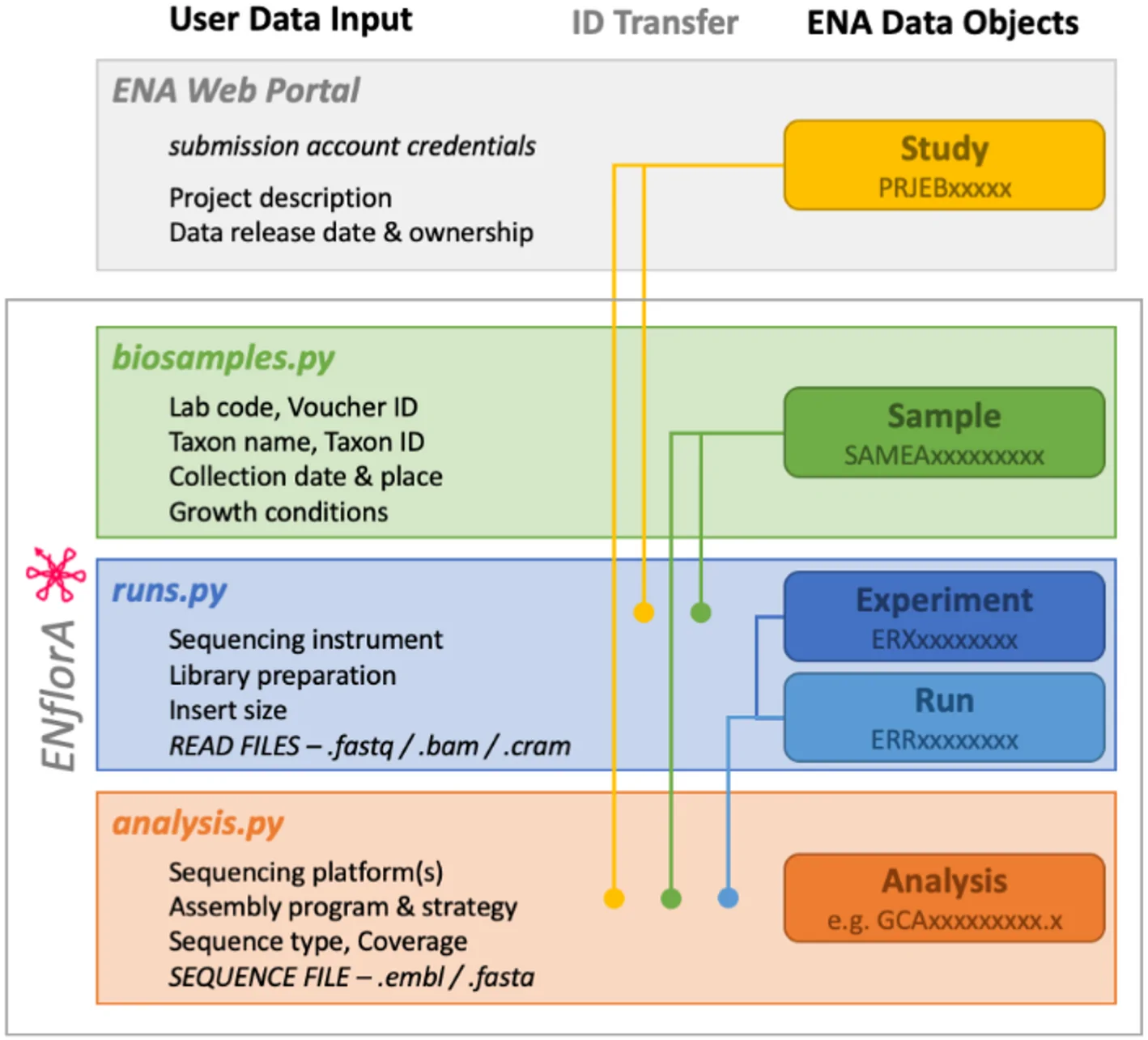
Overview of ENA data objects, including metadata requirements for each and application for the ENflorA scripts. Lines indicate which ENA accession numbers need to be handed over between submission steps (ID Transfer), ensuring traceability of data belonging to the same project or derived from the same source (sample / read files)

In Europe, DNA sequence data is mainly brokered and maintained through ENA, a European Molecular Biology Lab (EMBL) infrastructure founded in 1980 and currently managed by the European Bioinformatics Institute (EBI). ENA submissions typically require consistent handling of multiple linked object types according to ENA’s own metadata model (Fig. 1), including “Biosamples”, raw read libraries (“Experiments”/”Runs”), and genome assemblies/annotations (“Analyses”), each with strict metadata requirements and validation constraints. The submission tools provided by ENA itself are dichotomous: the online Webin Portal offers an intuitive graphical interface which is, however, functionally unscalable for bulk operations, leading to repetitive manual metadata entry and high error rates. Conversely, the Webin-CLI tool offers a robust validation environment suitable for bulk data, but requires the user to programmatically generate structured manifest files for every single data object, a task that necessitates scripting skills and thus also leaves a significant part of data curation with the user.

While large sequencing centers or projects may have bespoke, automated pipelines to push data to INSDC nodes [13], smaller or geographically distributed research groups often struggle with the technical complexity of submission interfaces. Platforms like COPO (Collaborative OPen Omics [14]) or GFBio (German Federation for Biological data [15]) have emerged to address this by providing centralized data brokering services. While powerful, such platforms typically require users to operate within specific web ecosystems and rely on external infrastructure and support, and may not be generally accessible. Alternatively, data analysis software may include INSDC submission functions, often targeting NCBI rather than ENA [16, 17], yet again be limited by cost, or a narrow specificity of purpose (Table 1). This may cause a significant “submission gap”: submission, and thus publication, may be significantly delayed, or existing data – especially metadata, large raw sequence files and further analysis results – lost through being omitted to “save time” or reduce effort [18, 19]. Thus, a “middleware” solution for sequence data submission is needed — a tool that runs locally on a user’s machine or High-Performance Computing (HPC) cluster, integrates with metadata handling in standard scientific collection and laboratory workflows via spreadsheets, and automates low-level interactions with ENA’s submission API even for bulk data without requiring the overhead of full platform adoption.

**Table 1:** Overview of publicly available ENA data submission tools

Name	Link	Ref	Data				1:1	First for	All taxa	Advantages	Limitations
webin-cli	https://github.com/enasequence/webin-cli	[20]	-	-	R	A	no	all sequence data	yes	ENA maintained	xml format expectations
COPO	https://github.com/EarlhamInst/COPO-production	[14]	P	S	R	A	no	plants	yes	webbased GUI	currently only for select projects*
ena-upload-cli	https://github.com/usegalaxy-eu/ena-upload-cli	[21]	P	S	R	-	no	SARS-CoV-2 viruses	yes	also for Galaxy, many additional functions	-
ENflorA	https://github.com/alfarodeprado/ENflorA	this article	-	S	R	A	no	plants	yes	Lightweight, builds on webin-cli	-
ena_submission	https://github.com/ukhsa-collaboration/ena_submission	-	P	S	R	-	yes	medicinal	yes	fully command-line	no ERC checklist for metadata
MAG2ENA	https://github.com/NFDI4Microbiota/MAG2ENA	- (in prep)	P	S	-	A	no	microbial genomes	no	-	no link to run data
seqsubmit	https://github.com/nf-core/seqsubmit/tree/dev	general: [22]	P	-	R	A	no	microbial genomes	(yes)	-	only previously submitted samples
MGnify	https://github.com/EBI-Metagenomics	general: [23]	-	-	-	A	no	microbial genomes	no	optional R integration	limited scope
q2-ena-uploader	https://github.com/bokulich-lab/q2-ena-uploader	[24]	P	S	R	-	yes	microbiome	(yes)	QIIME integration	unusual platform for other taxa

Data – ENA data types which can be created through the software, Bioprojects (P), Biosamples (S), Run/Experiment (R) and Analysis (A); 1:1 – the software expects a bijective relationship between the created datatypes (e.g. one Run per Biosample only) or cannot reference already databased objects; First for – taxon for which the software was initially developed; All taxa – the software can be used for all taxa, (yes) means in theory. * see https://copo-docs.readthedocs.io/en/latest/about/project-affiliations.html

The new software ENflorA was developed to help close the submission gap for NGS data, especially, yet not exclusively, for medium-sized projects without access to custom-made, in-house submission pipelines. It provides a lightweight, script-based workflow that standardizes the conversion of user tables into ENA biosample, run/experiment and analysis submission artifacts. Its design is pragmatic: it automates the data-intense submission steps (i.e. not the Bioproject creation itself, which is only required once per project and more easily done through the ENA web portal), keeps the user interface familiar (Excel/TSV tables), enforces schema constraints early, and automatically handles repeated steps such as staging, compression, manifest generation, and structured logging. The result is a small toolkit that helps research groups move from “metadata and sequence files” somewhere in their local research data storage, to a “submission-ready data package” with a reproducible, auditable process.

## Implementation

ENflorA is implemented as a modular suite of Python 3 scripts, designed to be platform-independent and easily deployable in diverse computing environments, from personal laptops running under Windows, macOS or Linux, to institutional HPC clusters using the slurm task manager. The tool consists of three primary modules: *biosamples.py*,* runs.py*, and *analysis.py*, which mirror ENA’s hierarchical object model (Fig. 1). While the typical use case would be the submission of first sample metadata (*biosamples.py*), then the associated read files (*runs.py*) and finally analysed data such as assembled plastid genomes (*analysis.py*) associated to a pre-existing Bioproject (created through the ENA Webin Portal), all three parts can be used independently. No bijective relationship between the handled data objects is assumed, which means that several analyses may reference the same read files, several read files or analyses can be associated with the same sample, and new read files or analyses can later be added to data objects already in the database. If two or more of the scripts are run consecutively, a helper script *(resolve_accessions.py)* can be used to translate project-internal lab codes and read file names used as temporary stand-ins in respective metadata files into the correct ENA accession numbers newly obtained in the previous submission step. Large files may be transferred through a more stable lftp connection.

### Environment management

To ensure reproducibility and ease of use, ENflorA includes a wrapper script that automates the initialization of its runtime environment. This wrapper handles the creation of a dedicated Python [25] virtual environment and installs necessary dependencies, including pandas [26] for data manipulation, pyyaml [27] for configuration management, openpyxl [28] for Excel support, and Biopython [29] for sequence formatting. Crucially, it also manages the retrieval and path configuration of the ENA Webin-CLI Java archive (.jar), ensuring that the user always validates against a compatible version of ENA’s data standards.

### Metadata parsing and validation

To date, most researchers and lab workflows store, or export, metadata in spreadsheets. ENflorA therefore uses this common and easy to manipulate format for metadata input, accepting both standard Excel (.xlsx) or tab-separated value (.tsv) files. ENflorA’s parsers expect specific column names that map to the corresponding ENA fields. For runs.py and analysis.py, these column names follow ENA’s own Webin-CLI manifest field names directly (e.g., STUDY, SAMPLE, INSTRUMENT, ASSEMBLY_TYPE), which are documented in the bundled template spreadsheets. For *biosamples.py*, the expected columns are implemented according to an ENA checklist specified in the config file, with mandatory and recommended fields enforced at the validation step; per default non-mandatory fields can additionally be set to mandatory in the config file, if required e.g. by the project or institutional policy, and pre-set values for specific fields or aliases for field names can be defined. Additional optional attributes accepted by ENA can be included by simply adding extra columns to the spreadsheet, which are then forwarded to ENA as supplementary sample attributes. ENflorA ships with the plant sample metadata checklist ERC000037 as its default/example; to submit data under a different ENA checklist, the respective .xml file must be downloaded first, and the helper script *make_table.py* run once to create the .xlsx template. The full list of ENA checklists is browsable online [9].

On invocation, each script performs a “pre-flight” validation of the associated metadata spreadsheet. This consists of two steps, syntax checking and file validation: first, it checks that all required columns are present and that mandatory fields are not empty. In *biosamples.py*, this includes parsing collection dates into ISO 8601 format (YYYY-MM-DD) from common alternatives like DD.MM.YYYY, and verifying that the ENA plant checklist’s mandatory fields are not empty; in *runs.py*, it checks that file type columns (FASTQ, BAM, CRAM) are present and consistent; in *analysis.py*, it verifies that exactly one of FLATFILE or FASTA is specified per row and that the assembly level is valid. Second, all scripts verify that referenced data files exist on the local disk and are readable.

### Module breakdown

The three ENflorA scripts are run independently, so as to incorporate data identifiers created during the previous step (Fig. 1). In consequence, the scripts may also be run individually, depending on the requirements of the project, e.g. if previously available read data already stored in ENA are reused to produce a new Analysis object (e.g. a plastome). Before the first script can be run, an ENA submission account is needed, and a Bioproject, with which all the newly uploaded data will be associated, needs to be created by hand. As Bioproject structure may be hierarchical, with several Primary submission projects grouped under an Umbrella project, and there is typically but a single Bioproject associated with many samples, read files or analyses, we chose not to include this step in ENflorA.

The first module, *biosamples.py*, manages the registration of biological source material to create *Biosample* objects. It generates the necessary XML submission files by mapping spreadsheet columns to ENA checklist attributes (e.g., geographic location, specimen voucher, collection date; based on an ENA sample metadata checklist). It supports batch registration, returning and logging the accession numbers (SAMEAxxxx) required for linking downstream data.

The second module, *runs.py*, handles raw read data and creates *Experiment* and *Run* objects in ENA. It triggers the automated validation and MD5 checksum calculation via the Webin-CLI for large FASTQ files, which is a mandatory step for ENA integrity verification. It constructs the Webin-CLI manifest files, defining the relationships between the Run, the Experiment (library strategy, source, sequencing instrument, selection), and the Sample, and returns the resulting accession numbers.

The third module, *analysis.py*, handles derived data, such as genome assemblies and annotations, creating ENA *Analysis* objects. Beyond the submission itself, it can also perform automated file conversion: ENA requires annotated assemblies to be submitted in the EMBL flatfile (.embl) format, which differs significantly from the GenBank (.gb) format put out by some sequence analysis tools (e.g. PlastidPipeline [30]). Through integration with Biopython, *analysis.py* can convert a Genbank file into a valid EMBL flat file, correctly mapping feature qualifiers to INSDC standards.

To make submission outputs easy to retrieve in both interactive and batch (HPC/SLURM) settings, all three modules write the accessions assigned by ENA into a plain-text file inside their respective submission folder (*biosample_accessions.txt*,* run_accessions.txt*,* analysis_accessions.txt*). These files are appended across runs and deduplicated, with test-server entries clearly flagged. The same accessions are also printed to standard output as they are returned by ENA, so they remain visible in SLURM .*out* log files. This separation between full submission logs (per-sample, verbose) and a flat accession registry (per-script, machine-readable) directly supports the next workflow step, where the previous object’s accession must be entered into the next spreadsheet.

### ID transfer

Linking data to its source, i.e. the underlying sample and/or read data, is an important feature for reproducibility and data FAIRness, and therefore rigorously required by our software. If multiple submission modules are run in succession, and the accessions for data objects created at the previous step are not yet known, entries in the field “isolate” (default ENA field for sample identifier) from the Biosamples metadata spreadsheet (e.g. BiosampleList.xlsx) can be used as a temporary replacement in the “SAMPLE” columns in the run and analysis metadata files (e.g. ExperimentList.xlsx, AnalysisList.xlsx) and “NAME” from the metadata file can substitute “RUN_REF” in the analysis metadata file. To translate these temporary IDs into the correct ENA accession numbers, the helper script *resolve_accessions.py* should be run between running the respective submission modules. However, as newly submitted data may be exclusively derived from previously existing ENA objects, or the relevant accessions may be found in files from multiple previous submissions, this script is not a default part of the submission process.

### Robust data transfer with lftp

Submitting terabytes of raw sequencing data over standard hypertext transfer protocol (HTTP) or basic file transfer protocol (FTP) connections is prone to failure due to network instability. ENflorA addresses this by integrating submission via lftp [31]: this robust FTP client supports automatic resumption of interrupted transfers. ENflorA includes a standalone helper script, independent from the three main Python modules, for lftp that mirrors the local staging directory to the ENA’s FTP server, webin2.ebi.ac.uk. This ensures that, if a connection drops e.g. during the overnight upload of a 100 GB dataset, the process can resume automatically from the last successful byte, rather than restarting from zero.

### Testing

Beside “live” submission, the three ENflorA scripts also allow testing the workflow and input data through “test” submission to the ENA Webin test server. From there, data is not made public, but automatically gets deleted after several days. To make this testing immediately accessible to new users, ENflorA includes a dedicated “--demo” mode: when any script is invoked with this flag, it uses bundled synthetic test data (biosample metadata, paired-end FASTQ reads, and both FASTA and EMBL flat-file assemblies) and submits them to the ENA test server, producing verbose logs that trace every step of the submission. This allows new users to end-to-end verify their entire setup, consisting of the credentials, the Python environment and the Java Webin-CLI, before preparing real data. The software can thus also be tested early on in a project before the actual project data arrives, e.g. by using already published data downloaded from ENA for a ‘dry run’. All sequences in the demo dataset are randomly generated and have no biological meaning; they are accepted by the ENA test server because they pass format validation, and test submissions are automatically deleted the next day from the test server.

We tested ENflorA on a variety of data, produced in several research projects at FU Berlin. This included a phylogeographic study on the medicinal plant *Arnica montana* [30] and a phylogenetic study on the Cunnoniaceae genus *Weinmannia* L [32]., involving both new and already databased samples / read data, as well as new raw sequences and plastome assemblies. Upload was tested both from local computers and FU Berlin’s HPC system Curta [33].

## Discussion

The rapid expansion of genomic research necessitates tools that can bridge the gap between data generation and data archiving. ENflorA addresses the specific needs of individuals, groups and small to medium-sized projects that generate high-value biodiversity data, but lack the dedicated bioinformatics engineering support of large sequencing centers.

By decoupling the definition of metadata (in spreadsheets) from the technical mechanics of submission (XML/manifest generation), ENflorA allows researchers to maintain control over their data, using familiar tools while ensuring compliance with INSDC standards. Unlike manual portal submissions, where users often omit optional metadata to save time, ENflorA’s template-based approach encourages the completion of rich metadata fields, such as geolocation, collection date, and specimen vouchers, before the submission process begins. This ensures that the resulting data is findable and reusable for future meta-analyses, contributing towards the FAIR data goal. Furthermore, by automating the conversion of annotation files (e.g., from GenBank format to ENA-compliant EMBL flatfiles) and reusing standard NCBI Taxonomy identifiers, the tool ensures a high level of interoperability.

In comparison to existing solutions, ENflorA occupies a unique niche: while Webin-CLI provides the “gold standard” for validation, it acts primarily as a validator and uploader rather than a workflow manager. By itself, it does not natively handle the batch logic required to link hundreds of samples to their respective runs and assemblies. Conversely, centralized platforms like COPO offer excellent wizard-driven interfaces but introduce dependencies on external servers and web-based workflows that may not align with local HPC data storage policies. ENflorA serves as a lightweight middleware that combines the batch-processing power of a command-line tool with the usability of a spreadsheet-driven workflow, running entirely within the user’s local infrastructure.

Its modular, yet compact design with one script per type of submission is an asset for projects working with different data sources: previously submitted samples and/or raw sequences can easily be referenced in the submission of “downstream” data types, and submitted alongside their counterparts resulting from new field or lab work. Equally, the scripts can easily be used by geographically distributed research projects, either for decentralized submission to the same project, or through gathering sequence files and format-compatible metadata spreadsheets from multiple groups for central submission by a data manager. It will thus significantly reduce the time spent on data deposition, freeing up resources e.g. for more thorough validation or to link additional metadata. The scripts can also be fully integrated into bioinformatic data analysis workflows, resulting in their better documentation and traceability.

While ENflorA’s “botanical” name reflects its origins, its architecture is fundamentally agnostic towards the (taxonomic) origin of the submitted data. The metadata requirements of *biosamples.py* can be adapted to any ENA metadata checklist. Submissions with *analysis.py* currently default to a single circular plastid chromosome for chromosome-level submissions, yet other types of assemblies/analyses may also be submitted by adapting the metadata file accordingly. However, additional types of analysis data which cannot be sumbitted through webin-cli, may require more profound changes or using another, more specialised tool.

## Conclusions

ENflorA provides a streamlined, user-friendly solution for the bulk submission of NGS data, effectively removing the technical barriers that often delay or discourage the deposition of genomic data into public repositories. By automating the translation of standard laboratory spreadsheets into ENA-compliant formats, the tool empowers researchers to adhere to FAIR data principles without requiring extensive bioinformatics expertise. As the volume of biodiversity data continues to grow, lightweight middleware tools like ENflorA will be essential for ensuring that this data is not only stored but remains discoverable, structured, and valuable to the global scientific community.

## Supplementary Information

Below is the link to the electronic supplementary material.

Supplementary Material 1

## Data Availability

The source code described in this article, including the synthetic test data, is available from https://github.com/alfarodeprado/ENflorA, and as a first release on Zenodo, https://doi.org/10.5281/zenodo.23441309. Project data used to test live upload is available from ENA, https://www.ebi.ac.uk/ena/, under the project IDs PRJEB89801 and PRJEB107803, and further IDs listed in Additional file A1.

## Code availability

Project name: ENflorA. Project home page: https://github.com/alfarodeprado/ENflorA. Operating system(s): Platform independent (tested on CentOS 7 Linux, Windows 11 and MacOS 12.7.6). Programming language: Python (primarily), Bash; Java required for Webin-CLI. Other requirements. Mandatory: Python 3.8+, pandas (≥ 1.2), PyYAML (≥ 5.0), openpyxl (≥ 3.0), Java 17+, ENA Webin-CLI (≥ 7.0; tested with 8.2.0). Optional: Biopython (≥ 1.78; .gb to .embl file format conversion), lftp (≥ 4.6; FTP-based upload), HPC with SLURM (file upload from HPC). License: MIT. Any restrictions to use by non-academics: None

## Abbreviations

API: Application Programming Interface
COPO: Collaborative OPen Omics
DDBJ: DNA Data Bank of Japan
EMBL: European Molecular Biology Laboratory
ENA: European Nucleotide Archive
FAIR: Findable, Accessible, Interoperable, Reusable
FTP: File Transfer Protocol
GFBio: German Federation for Biological data
HPC: High-Performance Computing
HTS: High-Throughput Sequencing
HTTP: Hypertext Transfer Protocol
INSDC: International Nucleotide Sequence Database Collaboration
JAR: Java Archive
NCBI: National Center for Biotechnology Information
NGS: Next-Generation Sequencing
TSV: Tab-Separated Values
WGS: Whole Genome Sequencing
XML: Extensible Markup Language
