# Supplementary material for: ENflorA: a flexible tool for the bulk submission of next generation sequencing data to the European Nucleotide Archive
Source: BMC Bioinformatics. 2026 Oct 21;27:242. doi: 10.1186/s12859-026-06714-7 (PMC13689177; doi:10.1186/s12859-026-06714-7)
Supplement: Supplementary file 1 [file 12859_2026_6714_MOESM1_ESM.pdf]

Additional File A1 – Project data uploaded with ENflorA

IDs of all data objects submitted with ENflorA to the European Nucleotide Archive (https://www.ebi.ac.uk/ena) in black. IDs in grey refer to previously uploaded data re-used for new analyses.

Bioproject ID and Reference	Biosample IDs	Run ID	Experiment ID	Assembly IDs	Species (Location)
**PRJEB89801**<br><br>*Arnica montana* chloroplast genome comparison (Annaldasula et al. 2025 [20])	SAMEA118348486 (ERS24618731)	ERR15302247	ERX14708563	GCA_965644005 (OZ286197.1)	*Arnica montana* (Mecklenburg-Western Pomerania)
	SAMEA118348487 (ERS24618732)	ERR15302248	ERX14708564	GCA_965643975 (OZ286200.1)	*Arnica montana* (Baden-Württemberg)
	SAMEA118771079 (ERS25262026)	ERR15310125	ERX14716541	GCA_965643905 (OZ286190.1)	*Arnica montana* (Brandenburg)
				GCA_965643905 (OZ286190.2)	
	SAMEA118771080 (ERS25262027)	ERR15336770	ERX14740391	GCA_965648725 (OZ287607.1)	*Arnica montana* (Galicia)
		ERR15337219	ERX14740840	GCA_965648765 (OZ287613.1)	
		ERR15340092	ERX14743793	GCA_965648695 (OZ287606.1)	
		ERR15340093	ERX14743794	GCA_965648755 (OZ287609.1)	
		ERR15340094	ERX14743795	GCA_965648715 (OZ287611.1)	
		ERR15340095	ERX14743796	GCA_965648745 (OZ287610.1)	
		ERR15340092 + ERR15340093 pp.	ERX14743793 + ERX14743794 pp.	GCA_965648705 (OZ287612.1)	
				GCA_965648735 (OZ287608.1)	
	SAMN23390517 (SRS11147526)	SRR17032105	SRX13222014	GCA_965362175 (OZ262470.1)	*Arnica montana* (Catalonia)
	SAMN16534177 (SRS7602750)	SRR12917857	SRX9382411	GCA_965643925 (OZ286192.1)	*Venegasia carpesioides* (California)
	SAMN23390523 (SRS11147532)	SRR17032099	SRX13222020	GCA_965643955 (OZ286199.1)	*Eupatorium cannabinum* (Catalonia)
	SAMN16534170 (SRS7602758)	SRR12917849	SRX9382419	GCA_965644015 (OZ286201.1)	*Guardiola tulocarpus* (Mexico)
	SAMEA9098631 (ERS6824762)	ERR8268641	ERX7831577	GCA_965643985 (OZ286198.1)	*Arnica montana* (Agder)
	SAMN15375640 (SRS7184476)	SRR12432532	SRX8928165	GCA_965643945 (OZ286194.1)	*Arnica montana* (Denmark)
	SAMEA117366522 (ERS22434057)	ERR14008899	ERX13410040	GCA_965644025 (OZ286202.1)	*Arnica montana* (Switzerland)
	SAMEA8202330 (ERS5888933)	ERR5554746	ERX5334788	GCA_965643995 (OZ286196.1)	*Arnica angustifolia* subsp. *alpina* (Finnmark)
	SAMEA8201770 (ERS5888373)	ERR5529436	ERX5309897	GCA_965643965 (OZ286195.1)	*Arnica griscomii* subsp. *frigida* (Alaska)
	SAMEA8201771 (ERS5888374)	ERR5529317	ERX5309778	GCA_965643915 (OZ286193.1)	*Arnica lessingii* (Alaska)
	SAMEA8201772 (ERS5888375)	ERR5529299	ERX5309760	GCA_965643935 (OZ286191.1)	*Arnica unalaschcensis* (Alaska)
**PRJEB107803**<br><br>Plastid genomes of *Weinmannia* (Fajardo-Gutierrez et al. in prep) embargoed until 2027-06-30	SAMEA122124705 (ERS29633294)	ERR16986347	ERX16370014	OZ457659.1	*Weinmannia epicae* (Bolivia)
	SAMEA122124704 (ERS29633293)	ERR16986346	ERX16370013	OZ457664.1	*Weinmannia rhoifolia* (Bolivia)
	SAMEA122124703 (ERS29633292)	ERR16986345	ERX16370012	OZ457665.1	*Weinmannia cochensis* (Peru)
	SAMEA122124702 (ERS29633291)	ERR16986344	ERX16370011	OZ457649.1	*Weinmannia polyphylla* (Ecuador)
	SAMEA122124701 (ERS29633290)	ERR16986343	ERX16370010	OZ457653.1	*Weinmannia bangii* (Bolivia)
	SAMEA122124700 (ERS29633289)	ERR16986342	ERX16370009	OZ457657.1	*Weinmannia* cf. *polyphylla* (Ecuador)
	SAMEA122124699 (ERS29633288)	ERR16986341	ERX16370008	OZ457663.1	*Weinmannia tinctoria* (Reunion)
	SAMEA122124698 (ERS29633287)	ERR16986340	ERX16370007	OZ457660.1	*Weinmannia trichosperma* (Chile)
	SAMEA122124697 (ERS29633286)	ERR16986339	ERX16370006	OZ457656.1	*Weinmannia trichosperma* (Chile)
	SAMEA122124696 (ERS29633285)	ERR16986338	ERX16370005	OZ457650.1	*Weinmannia tinctoria* (Reunion)
	SAMEA122124695 (ERS29633284)	ERR16986337	ERX16370004	OZ457651.1	*Weinmannia mauritiana* (Reunion)
	SAMEA122124694 (ERS29633283)	ERR16986336	ERX16370003	OZ457654.1	*Weinmannia lechleriana* (Bolivia)
	SAMEA122124693 (ERS29633282)	ERR16986335	ERX16370002	OZ457661.1	*Weinmannia pentaphylla* (Ecuador)

**Bioproject ID and Reference**	**Biosample IDs**	**Run ID**	**Experiment ID**	**Assembly IDs**	**Species** (Location)
	SAMEA122124692 (ERS29633281)	ERR16986334	ERX16370001	OZ457655.1	*Weinmannia costulata* (Ecuador)
	SAMEA122124691 (ERS29633280)	ERR16986333	ERX16370000	OZ457652.1	*Pterophylla rutenbergii* (Madagascar)
	SAMEA122124690 (ERS29633279)	ERR16986332	ERX16369999	OZ457658.1	*Weinmannia boliviensis* (Bolivia)
	SAMEA122124689 (ERS29633278)	ERR16986331	ERX16369998	OZ457662.1	*Weinmannia subsessiliflora* subsp. *caquetana* (Bolivia)
	SAMEA122124688 (ERS29633277)	ERR16986330	ERX16369997	OZ457668.1	*Weinmannia reticulata* (Bolivia)
	SAMEA122124687 (ERS29633276)	ERR16986329	ERX16369996	OZ457666.1	*Weinmannia boliviensis* (Bolivia)
	SAMEA122124686 (ERS29633275)	ERR16986328	ERX16369995	OZ457673.1	*Weinmannia microphylla* (Bolivia)
	SAMEA122124685 (ERS29633274)	ERR16986327	ERX16369994	OZ457672.1	*Weinmannia rhoifolia* (Peru)
	SAMEA122124684 (ERS29633273)	ERR16986326	ERX16369993	OZ457670.1	*Weinmannia fagaroides* (Peru)
	SAMEA122124683 (ERS29633272)	ERR16986325	ERX16369992	OZ457667.1	*Weinmannia haenkeana* (Bolivia)
	SAMEA122124682 (ERS29633271)	ERR16986324	ERX16369991	OZ457669.1	*Weinmannia cundinamarcensis* (Colombia)
	SAMEA122124681 (ERS29633270)	ERR16986323	ERX16369990	OZ457671.1	*Weinmannia* aff. *tomentosa* (Colombia)
	SAMEA122124680 (ERS29633269)	ERR16986322	ERX16369989	OZ457648.1	*Weinmannia tomentosa* (Colombia)
	SAMEA122124679 (ERS29633268)	ERR16986321	ERX16369988	OZ457636.1	*Weinmannia auriculata* (Colombia)
	SAMEA122124678 (ERS29633267)	ERR16986320	ERX16369987	OZ457630.1	*Cunonia macrophylla* (New Caledonia)
	SAMEA122124677 (ERS29633266)	ERR16986319	ERX16369986	OZ457641.1	*Weinmannia boliviensis* (Bolivia)
	SAMEA122124676 (ERS29633265)	ERR16986318	ERX16369985	OZ457635.1	*Weinmannia* cf. *microphylla* (Bolivia)
	SAMEA122124675 (ERS29633264)	ERR16986317	ERX16369984	OZ457639.1	*Weinmannia trollii* (Bolivia)
	SAMEA122124674 (ERS29633263)	ERR16986316	ERX16369983	OZ457632.1	*Weinmannia spruceana* (Ecuador)
	SAMEA122124673 (ERS29633262)	ERR16986315	ERX16369982	OZ457637.1	*Weinmannia costulata* (Ecuador)
	SAMEA122124672 (ERS29633261)	ERR16986314	ERX16369981	OZ457631.1	*Weinmannia latifolia* (Ecuador)
	SAMEA122124671 (ERS29633260)	ERR16986313	ERX16369980	OZ457634.1	*Weinmannia elliptica* (Ecuador)
	SAMEA122124670 (ERS29633259)	ERR16986312	ERX16369979	OZ457643.1	*Weinmannia aff. cochensis* (Ecuador)
	SAMEA122124669 (ERS29633258)	ERR16986311	ERX16369978	OZ457640.1	*Weinmannia mariquitae* (Ecuador)
	SAMEA122124668 (ERS29633257)	ERR16986310	ERX16369977	OZ457644.1	*Weinmannia mariquitae* (Colombia)
	SAMEA122124667 (ERS29633256)	ERR16986309	ERX16369976	OZ457638.1	*Weinmannia burserifolia* (Panama)
	SAMEA122124666 (ERS29633255)	ERR16986308	ERX16369975	OZ457647.1	*Weinmannia microphylla* (Bolivia)
	SAMEA122124665 (ERS29633254)	ERR16986307	ERX16369974	OZ457646.1	*Weinmannia bangii* (Bolivia)
	SAMEA122124664 (ERS29633253)	ERR16986306	ERX16369973	OZ457642.1	*Weinmannia auriculifera* (Bolivia)
	SAMEA122124663 (ERS29633252)	ERR16986305	ERX16369972	OZ457645.1	*Weinmannia* x *yungasensis* (Bolivia)
	SAMEA122124662 (ERS29633251)	ERR16986304	ERX16369971	OZ457627.1	*Weinmannia* cf. *lentiscifolia* (Colombia)
	SAMEA122124661 (ERS29633250)	ERR16986303	ERX16369970	OZ457629.1	*Weinmannia* x *macrophylla* (Colombia)
	SAMEA122124660 (ERS29633249)	ERR16986302	ERX16369969	OZ457626.1	*Weinmannia tomentosa* (Colombia)
	SAMEA122124659 (ERS29633248)	ERR16986301	ERX16369968	OZ457633.1	*Weinmannia auriculata* (Colombia)
	SAMEA122124658 (ERS29633247)	ERR16986300	ERX16369967	OZ457625.1	*Weinmannia fagaroides* (Colombia)
	SAMEA122124657 (ERS29633246)	ERR16986299	ERX16369966	OZ457628.1	*Weinmannia rollottii* (Colombia)
	SAMEA122124656 (ERS29633245)	ERR16986298	ERX16369965	OZ457624.1	*Weinmannia ovata* (Colombia)
